# Dimer‐Specific FokT‐seq Reveals DNA‐Binding Dimerization and Novel Genomic Targets of TDP‐43

**DOI:** 10.1002/advs.202508902

**Published:** 2025-08-23

**Authors:** Mingming Yang, Qi Wang, Ruolan Yan, Xiaochuan Wang, Jianlan Gu

**Affiliations:** ^1^ Department of Pathophysiology School of Basic Medicine Key Laboratory of Education Ministry/Hubei Province of China for Neurological Disorders Tongji Medical College Huazhong University of Science and Technology Wuhan 430030 China; ^2^ Departmentof Biochemistry and Molecular Biology, School of Medicine, Key Laboratory of Neuroregeneration and Ministry of Education of Jiangsu, Co‐innovation Center of Neuroregeneration Nantong University Nantong 226001 China; ^3^ Hubei Key Laboratory of Cognitive and Affective Disorders, Institute of Biomedical Sciences, School of Medicine Jianghan University Wuhan 430056 China

**Keywords:** dimerization, DNA binding, FokT‐seq, TDP‐43

## Abstract

In postmortem brain tissues of patients with sporadic amyotrophic lateral sclerosis (ALS), the dimerization ability of TAR DNA‐binding protein 43 (TDP‐43) is impaired, accompanied by an accumulation of insoluble TDP‐43. Thus, the loss of TDP‐43 dimerization may play a critical driving role in ALS pathogenesis, although its underlying mechanism remains unclear. In this study, the FokT (FokI‐TDP‐43) system is developed, which fuses TDP‐43 protein with FokI nuclease. By restoring TDP‐43 dimerization, this system reactivates FokI nuclease activity, enabling the cleavage of DNA targets bound by TDP‐43. Additionally, the FokT‐seq (FokT combined with genome‐wide unbiased identification of DNA double‐strand breaks enabled by sequencing, Guide‐seq) method is established, allowing genome‐wide detection of DNA sites bound by dimerized TDP‐43. These findings reveal the essential role of TDP‐43 dimerization in DNA binding, identify a series of related targets. Furthermore, this study offers a powerful tool for investigating dimerized transcription factors.

## Graphical abstract

Loss of TDP‐43 dimerization is implicated in ALS, yet its mechanistic role remains elusive. This study introduces FokT, a novel system that restores TDP‐43 dimerization to map its DNA targets via FokT‐seq. The findings uncover key binding sites and offer a versatile tool for exploring dimer‐dependent transcriptional regulation.

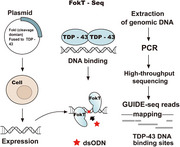

## 1 Introduction

Transactive response DNA binding protein 43 (TDP‐43) is a multifunctional RNA/DNA‐binding protein that plays a central role in nuclear RNA metabolism, including pre‐mRNA splicing, mRNA stability regulation, and transport.^[^ ^1^, ^2^, ^3^, ^4^, ^5^, ^6^, ^7^ ^]^ Its dysfunction—particularly cytoplasmic mislocalization and aggregation—is a defining hallmark associated with several neurodegenerative diseases, most notably amyotrophic lateral sclerosis (ALS) and frontotemporal lobar degeneration (FTLD).^[^ ^8^, ^9^, ^10^, ^11^ ^]^ While inclusions of TDP‐43 have been widely reported in disease conditions, the structural mechanisms driving its transition from a soluble, functional state to an insoluble, aggregated form have long remained unclear. In recent years, accumulating evidence has revealed that the dimerization or oligomerization capacity of TDP‐43 constitutes a fundamental structural basis for both its physiological function and its resistance to pathological aggregation. The N‐terminal domain (NTD) of TDP‐43 mediates head‐to‐tail interactions that drive the formation of dynamic and reversible functional oligomers. Afroz et al.^[^ ^12^ ^]^ demonstrated through structural and functional analyses that NTD‐driven oligomerization is widespread in the physiological nuclear environment and is essential for the protein's RNA splicing activity. Furthermore, such oligomeric organization prevents aberrant aggregation driven by its C‐terminal low‐complexity domain (LCD). This mechanism was further supported by mutational and functional studies. Wang et al.^[^ ^13^ ^]^ introduced a phosphomimetic mutation at serine 48 (TDP‐43^S48E^) to disrupt NTD‐mediated dimerization/oligomerization, resulting in impaired liquid‐liquid phase separation (LLPS) and severely compromised splicing function, indicating that the oligomeric state is crucial for the biological activity of TDP‐43. Perez‐Berlanga et al.^[^ ^14^ ^]^ systematically dissected the roles of oligomerization and RNA binding, showing that loss of oligomerization leads to cytoplasmic mislocalization and aggresome‐dependent inclusion formation, while loss of RNA binding promotes nuclear aggregation through LLPS. These findings underscore distinct and separable pathological pathways arising from different functional impairments of TDP‐43.

More direct evidence was provided by Oiwa et al.,^[^ ^15^ ^]^ who analyzed brain tissues from ALS patients and observed a significant reduction in dimeric TDP‐43 accompanied by an increase in its monomeric form. The authors proposed that TDP‐43 monomerization is a key determinant for initiating pathological aggregation. Using the TDP‐DiLuc reporter system, they demonstrated in real time that loss of oligomerization precedes inclusion formation, providing the first direct link between impaired oligomeric structure and pathological aggregation in human tissues. Collectively, these studies establish that TDP‐43 oligomerization is not only essential for its physiological function but also serves as the first line of defense against abnormal aggregation or aggregation associated with disease. Disruption of this structural regulation markedly increases the protein's propensity to aggregate and drives the formation of inclusions implicated in TDP‐43 proteinopathies, paticularly ALS. Although its role in disease pathology is becoming increasingly clear, the precise molecular mechanisms governing TDP‐43 oligomerization under physiological conditions remain to be fully elucidated. Understanding this process is critical for deciphering ALS pathogenesis and may inform the development of therapeutic strategies targeting the stabilization of TDP‐43 in its functional oligomeric state.

FokI is a Type II restriction endonuclease consisting of a DNA recognition domain and a catalytic domain.^[^ ^16^, ^17^, ^18^ ^]^ FokI itself lacks intrinsic DNA‐binding specificity, and its cleavage activity is only activated upon dimerization. This property has made FokI a valuable tool in genome editing; for instance, zinc finger nucleases (ZFNs) and transcription activator‐like effector nucleases (TALENs) are based on FokI and are designed to specifically cleave target DNA sequences.^[^ ^19^, ^20^, ^21^, ^22^ ^]^ Leveraging this unique feature of FokI, we developed an innovative FokT system by fusing the catalytic domain of FokI with TDP‐43, making the dimerization of TDP‐43 a trigger for FokI cleavage activity. When TDP‐43 dimerizes and binds specific DNA targets, the catalytic function of FokI is activated, leading to double‐strand breaks (DSBs) at TDP‐43‐bound DNA sites. However, when TDP‐43 dimerization is disrupted (e.g., in the FokT^N6A^ mutant), this functionality is entirely lost.^[^ ^14^, ^15^ ^]^

To investigate the physiological role of TDP‐43 dimerization, we conducted a comprehensive analysis of the downstream effects of the FokT system by integrating transcriptomic sequencing technologies. Our results demonstrated that the transcriptional changes observed upon TDP‐43 knockdown closely mirrored those induced by FokT system overexpression, suggesting that TDP‐43 primarily regulates gene transcription through interaction with DNA, with dimerization playing a key role in this process. Furthermore, we employed the FokT‐induced DSB strategy, introducing double‐stranded oligonucleotides to precisely map downstream DNA targets bound by dimerized TDP‐43. For the first time, this approach unveiled a genome‐wide map of TDP‐43 dimerization‐dependent DNA‐binding sites.

In summary, this study uncovers a novel transcriptional regulatory function of TDP‐43 beyond its canonical role in RNA splicing, showing that its dimerization facilitates downstream gene transcription regulation through direct binding to specific DNA. This finding significantly expands our understanding of the function of TDP‐43 and highlights the potential role of its dimerization in TDP‐43 proteinopathies, like ALS. Additionally, the FokT‐seq method provides a universal and efficient tool for studying the DNA‐binding mechanisms of other dimerization‐dependent proteins, laying a solid foundation for basic research and the development of therapeutic strategies for related diseases.

## 2 Results

### 2.1 FokT System: An Innovative Tool for Precisely Capturing the Dynamic Dimerization of TDP‐43

The dimerization of TDP‐43 is highly dynamic, and this dynamic property makes it challenging to capture a stable dimerization structure using traditional structural biology methods, such as X‐ray crystallography or nuclear magnetic resonance (NMR).^[^ ^12^ ^]^ However, understanding the role of TDP‐43 dimerization in neurodegenerative diseases is crucial for uncovering the mechanisms underlying ALS and related disorders. We hypothesize that TDP‐43 dimerization not only influences its regulatory role in RNA splicing,^[^ ^12^ ^]^ but may also participate in transcriptional regulation by modulating its interaction with DNA. Nevertheless, studying TDP‐43 dimerization presents significant technical challenges. For instance, traditional biochemical assays struggle to distinguish between the specific functional mechanisms of TDP‐43 monomers and dimers. These challenges have limited our understanding of the functional roles of TDP‐43 dimerization.

**Figure 1:**
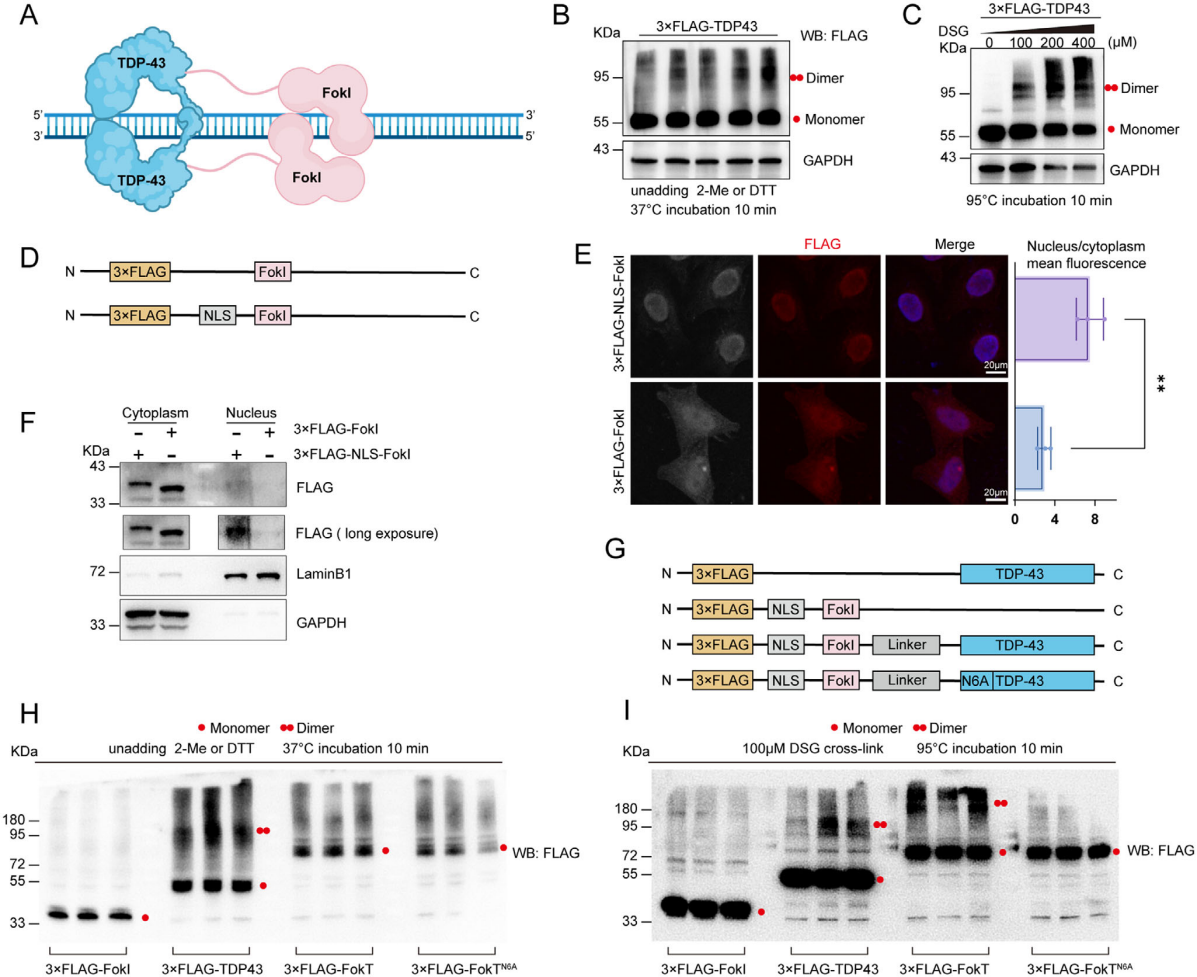
The dimerization of FokI‐TDP43 is dependent on the dimerization of TDP‐43. **A**) Conceptual schematic of the FokT design: dimerized FokT cleaving double‐stranded DNA. **B**) Non‐reducing electrophoresis of 293T cells overexpressing the 3×FLAG‐TDP‐43 plasmid shows the presence of both monomeric and dimeric TDP‐43 under physiological conditions. GAPDH was used as the loading control. **C**) Crosslinking of 3×FLAG‐TDP‐43 in 293T cells with increasing concentrations of DSG shows an increase in the dimeric form and a decrease in the monomeric form. GAPDH was used as the loading control. **D**) Schematic representation of the designs of 3×FLAG‐FokI and 3×FLAG‐NLS‐FokI plasmids. **E**) Representative images of the nuclear and cytoplasmic localization of 3×FLAG‐FokI and 3×FLAG‐NLS‐FokI in 293T cells and the quantification of nucleus/cytoplasm mean fluorscence. Data are presented as mean ± S.E.M., and analyzed by two‐tailed *t* test. (n=6. *, FokI versus NLS‐FokI; ** *p*≤0.01). **F**) Nuclear and cytoplasmic fractionation analysis shows that 3×FLAG‐FokI is localized exclusively in the cytoplasm, while 3×FLAG‐NLS‐FokI is present in both the cytoplasm and nucleus. Lamin B1 was used as the nuclear marker, and GAPDH was used as the cytoplasmic marker. **G**) Schematic representation of 3×FLAG‐TDP‐43, 3×FLAG‐NLS‐FokI, 3×FLAG‐NLS‐FokT, and 3×FLAG‐NLS‐FokT^N6A^ plasmid constructs. **H**) Non‐reducing electrophoresis of 293T cells overexpressing 3×FLAG‐TDP‐43, 3×FLAG‐NLS‐FokI, 3×FLAG‐NLS‐FokT, and 3×FLAG‐NLS‐FokT^N6A^ plasmids. The FLAG antibody was used to detect their dimeric forms. **I**) Overexpression of 3×FLAG‐TDP‐43, 3×FLAG‐NLS‐FokI, 3×FLAG‐NLS‐FokT, and 3×FLAG‐NLS‐FokT^N6A^ plasmids in 293T cells followed by crosslinking with 100 µM DSG. Western blot analysis was performed to examine their dimerization.

To overcome these difficulties, we developed an innovative solution. By fusing the catalytic domain of FokI with TDP‐43, we constructed the FokT system. FokI is an enzyme that requires dimerization to activate its cleavage activity, making the FokT system a suitable tool for investigating the function of TDP‐43 dimerization. In theory, when TDP‐43 dimerizes and binds to DNA, the catalytic activity of FokI is activated, resulting in the cleavage of DNA bound by TDP‐43 (Figure **1**A).

We first validated the ability of TDP‐43 to dimerize under normal conditions using non‐reducing electrophoresis (Figure 1B). Additionally, crosslinking experiments using varying concentrations of disuccinimidyl glutarate (DSG) demonstrated that TDP‐43 dimerization significantly increased with higher DSG concentrations, further confirming its dimerization capability (Figure 1C). Since unmodified FokI is primarily localized in the cytoplasm, we incorporated a nuclear localization signal (NLS) into its structure to ensure its nuclear entry (Figure 1D). Immunofluorescence assays showed that FokI localization in the nucleus was significantly enhanced after adding the NLS, and nuclear‐cytoplasmic fractionation experiments confirmed that FokI without the NLS was almost entirely excluded from the nucleus (Figure 1E,F).

Next, we constructed expression vectors for TDP‐43, FokT, and a mutant FokT^N6A^ that disrupts the TDP‐43 dimerization binding site (Figure 1G). Non‐reducing electrophoresis was used to examine whether the FokT system could dimerize. The results showed that the catalytic domain of FokI alone could not form dimers, whereas FokT, fused with TDP‐43, demonstrated clear dimerization (Figure 1H). Furthermore, when the N‐terminal dimerization binding site of TDP‐43 was disrupted, the dimerization capacity of FokT^N6A^ was significantly reduced. To further validate these findings, we performed DSG crosslinking experiments again and found that FokT formed distinct dimers after crosslinking, while FokT^N6A^ showed no significant dimerization (Figure 1I).

In summary, these results clearly demonstrate that the FokT system we developed can achieve dimerization, and this dimerization depends on the dimerization properties of TDP‐43. The FokT system provides a powerful tool for investigating the functional roles of TDP‐43 dimerization and its involvement in TDP‐43 proteinopathies.

### 2.2 The Key to FokT System's DNA Cleavage Lies in the N‐Terminal Dimerization Function of TDP‐43

**Figure 2:**
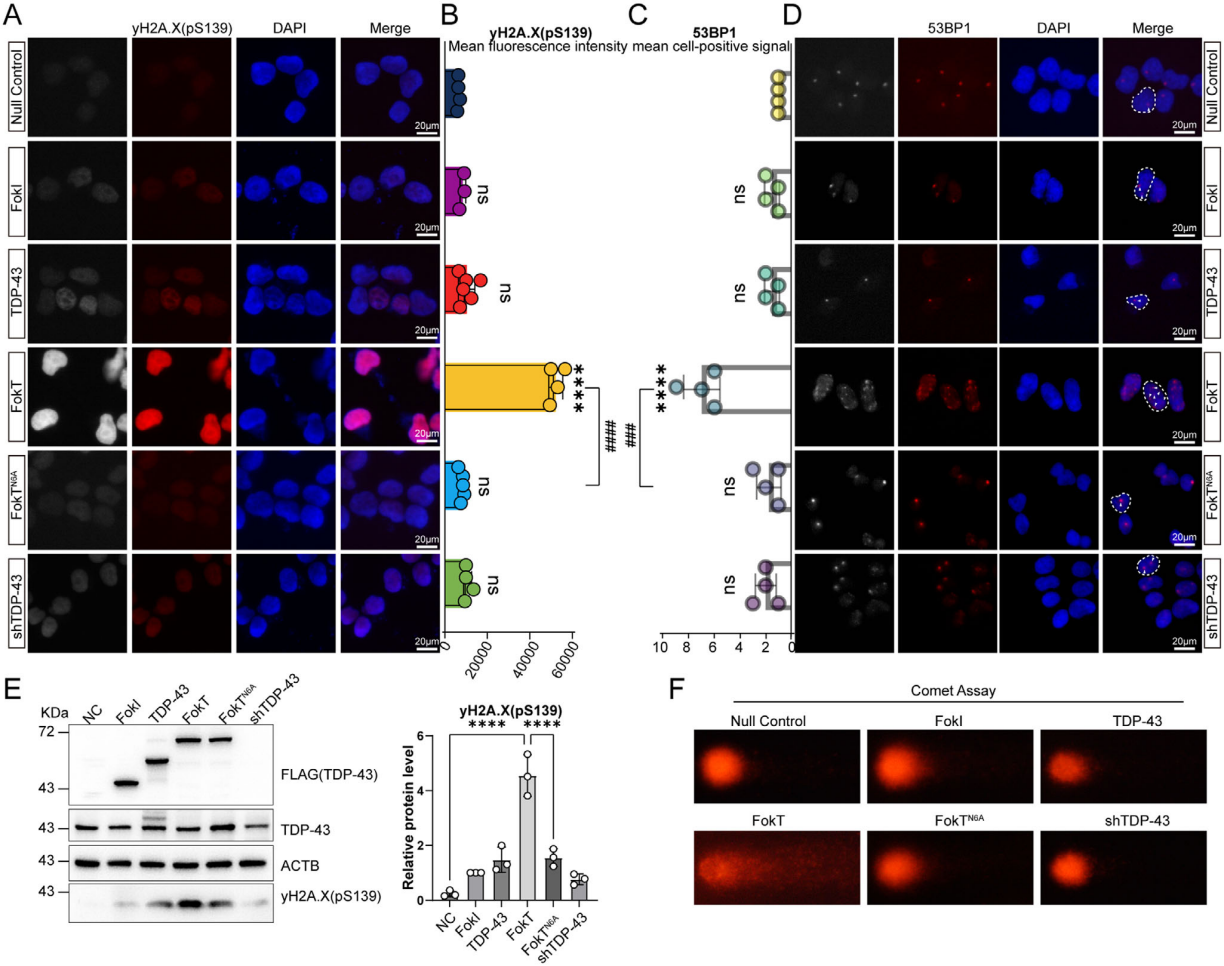
FokT mediates DNA cleavage, whereas FokT^N6A^ does not. A) Representative fluorescence images of γH2A.X (pS139, red) in 293T cells overexpressing 3×FLAG‐TDP‐43, 3×FLAG‐NLS‐FokI, 3×FLAG‐NLS‐FokT, and 3×FLAG‐NLS‐FokT^N6A^ plasmids. **B**) Quantification of γH2A.X (pS139) immunofluorescence intensity shown in panel a (n = 4., *, treatment group versus Null Control; ^#^, FokT versus FokT^N6A^; ****p≤ 0.0001, ^####^ *p*≤ 0.0001, ns, no significance). **C**) Quantification of the average number of 53BP1 foci (red) per nucleus shown in panel d (n = 4. *, treatment group versus Null Control; ^#^, FokT versus FokT^N6A^; **** *p*≤ 0.0001, ^###^ *p*≤ 0.001. ns, no significance). **D**) Representative fluorescence images of 53BP1 (red) in 293T cells overexpressing 3×FLAG‐TDP‐43, 3×FLAG‐NLS‐FokI, 3×FLAG‐NLS‐FokT, and 3×FLAG‐NLS‐FokT^N6A^ plasmids. **E**) Western blot analysis of γH2A.X (pS139) expression in 293T cells overexpressing 3×FLAG‐TDP‐43, 3×FLAG‐NLS‐FokI, 3×FLAG‐NLS‐FokT, and 3×FLAG‐NLS‐FokT^N6A^ plasmids. ACTB was used as the loading control. The bar graph represents quantification of γH2A.X (pS139) expression based on Western blot results (n = 3. *, NC or FokT versus FokT^N6A;^ ****p≤ 0.0001). **F**) DNA damage assessment in 293T cells overexpressing 3×FLAG‐TDP‐43, 3×FLAG‐NLS‐FokI, 3×FLAG‐NLS‐FokT, and 3×FLAG‐NLS‐FokT^N6A^ plasmids using comet assays. Longer tail lengths indicate greater DNA damage. Data are presented as mean ± S.E.M., and analyzed by One‐way ANOVA.

After confirming that FokT can dimerize, we further investigated whether FokT possesses DNA cleavage activity. To this end, we transfected 293T cells with plasmids encoding FokI, TDP‐43, FokT, FokT^N6A^, and shTDP‐43, respectively, to assess their DNA cleavage capability. Subsequently, we performed immunofluorescence to detect the expression of DNA damage markers γH2AX (pS139) and 53BP1^[^ ^23^, ^24^ ^]^ (Figure **2**A,D). The results showed that in 293T cells transfected with FokT, γH2AX (pS139) and 53BP1 exhibited significantly enhanced expression, with strong positive signals observed (Figure 2B,C). However, no such phenomenon was observed in cells transfected with FokT^N6A^. These findings indicate that FokT is indeed capable of cleaving double‐stranded DNA, whereas FokT^N6A^ cannot activate DNA cleavage due to the disruption of the N‐terminal dimerization binding site of TDP‐43.

To further validate this observation, we used Western blotting to detect the expression levels of γH2AX (pS139). Consistent with the immunofluorescence results, γH2AX (pS139) protein expression was significantly increased in cells transfected with FokT, while no significant changes were observed in the FokT^N6A^ group (Figure 2E).

Additionally, we performed comet assays to further confirm DNA cleavage. The results showed that 293T cells transfected with FokT exhibited prominent comet tailing, indicative of DNA breaks, whereas no tailing was observed in cells transfected with FokT^N6A^ (Figure 2F). This further corroborates that FokT effectively cleaves DNA, and this activity depends on the N‐terminal dimerization functionality of TDP‐43.

In summary, these results unequivocally demonstrate that FokT has the ability to cleave DNA, and this cleavage activity is entirely dependent on the N‐terminal dimerization domain of TDP‐43. These findings provide a robust experimental basis for further studies on the role of TDP‐43 dimerization in DNA binding and gene regulation.

### 2.3 Transcriptomics Reveals High Consistency Between FokT and TDP‐43 Knockdown Effects

**Figure 3:**
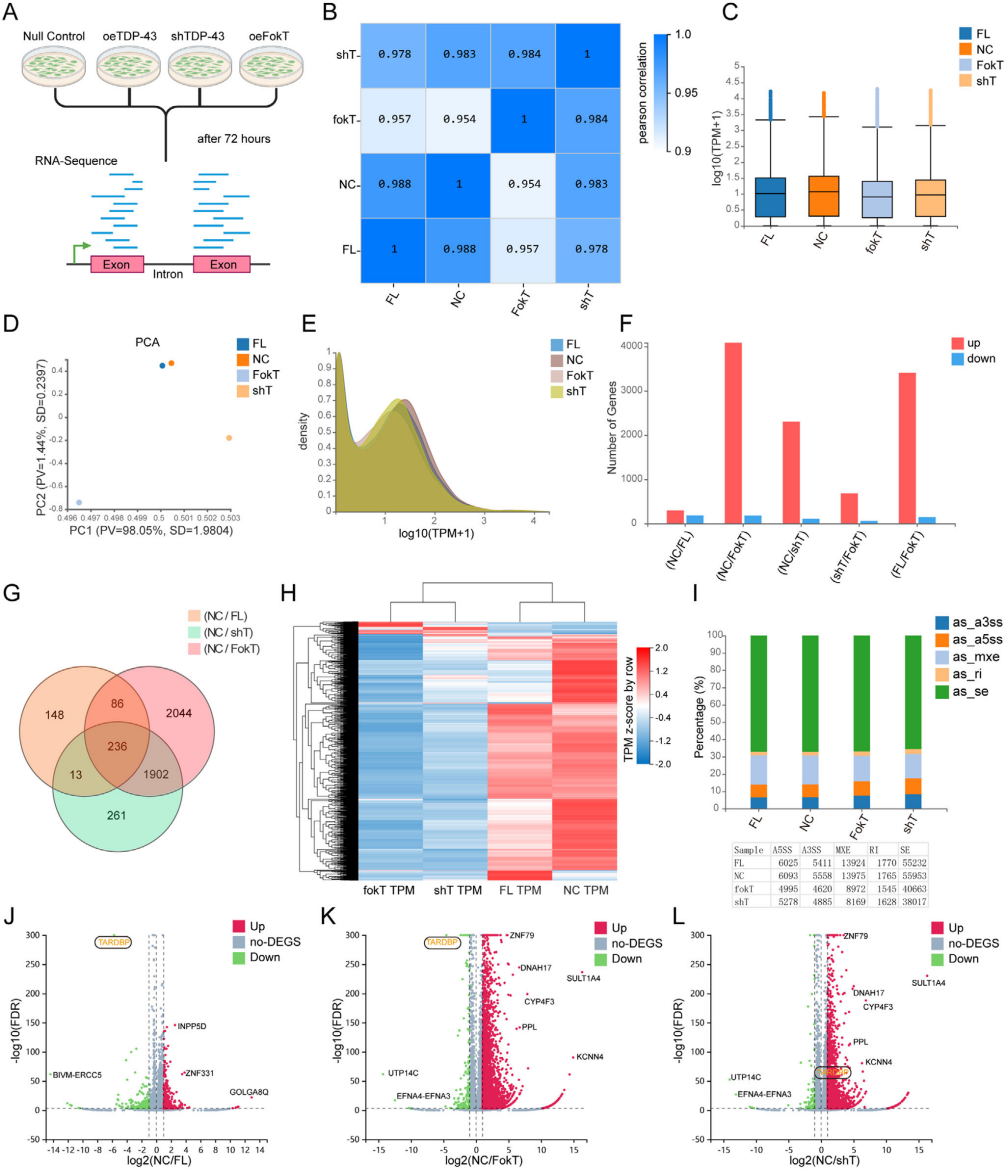
FokT precisely cleaves downstream genes and recapitulates TDP‐43 knockdown effects. **A**) RNA was extracted from 293T cells after 72 h of overexpression with 3×FLAG‐TDP‐43, 3×FLAG‐NLS‐FokT, and shTDP‐43 plasmids, followed by RNA‐Seq analysis. **B**) Pearson correlation coefficients for gene expression levels were calculated between all pairs of samples and visualized as a heatmap. The correlation coefficients reflect the overall similarity in gene expression profiles between samples, with higher coefficients indicating greater similarity. **C**) Boxplots display the distribution of gene expression levels for each sample. The X‐axis represents sample names, and the Y‐axis represents log10(TPM+1). Each boxplot illustrates five statistics (from top to bottom: maximum, upper quartile, median, lower quartile, and minimum, excluding outliers). **D**) Principal component analysis (PCA) plot showing the first principal component (PC1) on the X‐axis and the second principal component (PC2) on the Y‐axis. Each point represents a sample, with colors indicating sample groups. PV represents the proportion of variance, and SD represents standard deviation. **E**) Density plots depict the distribution of gene expression levels within each sample, showing the range of expression values where gene abundance is concentrated. The X‐axis represents log10(TPM+1), and the Y‐axis represents gene density, which is the proportion of genes at a given expression level relative to the total number of expressed genes. **F**) Bar graph showing the number of differentially expressed genes (DEGs) for each comparison group. The X‐axis represents comparison groups, and the Y‐axis represents DEG counts. Red bars indicate upregulated DEGs, while blue bars indicate downregulated DEGs. **G**) Venn diagram illustrating the overlap and uniqueness of gene sets. Each circle represents a gene set, with overlapping areas indicating shared genes among sets. Numbers within each area represent the count of genes. **H**) Heatmap of gene expression normalized by z‐scores (row direction). The X‐axis represents the log2‐transformed expression values, and the Y‐axis represents genes. In the default color scheme, red indicates high expression levels, and blue indicates low expression levels. **I**) Stacked bar chart summarizing the distribution of alternative splicing events in each comparison group. The X‐axis represents comparison groups, and the Y‐axis represents the proportion of each alternative splicing type. Different colors correspond to different splicing event types. **J–L**) Volcano plots displaying differentially expressed genes. The X‐axis represents log2 fold changes, and the Y‐axis represents ‐log10 *p*‐values. Red dots indicate upregulated DEGs, blue dots indicate downregulated DEGs, and gray dots indicate non‐DEGs.

To verify whether FokT cleaves downstream DNA targets of TDP‐43, we transfected 293T cells with plasmids encoding TDP‐43, FokT, and shTDP‐43, extracted total RNA after 48 h of culture, and performed transcriptome sequencing (Figure **3**A). The results showed that the transcriptomes of the FokT and shTDP‐43 groups shared a similarity of 98.4% (Figure 3B), with highly similar distributions of gene expression levels. Box plot analysis indicated that the degree of data dispersion was also highly consistent between the two groups (Figure 3C). Principal component analysis (PCA) further supported the high similarity between the FokT and shTDP‐43 groups (Figure 3D). Density plots and stacked bar charts also revealed that the gene expression levels in the FokT and shTDP‐43 groups were concentrated within the same range (Figure 3E).

In the differential gene expression analysis, bar chart results showed that FokT caused more gene downregulation than the shTDP‐43 group (Figure 3F). Through Venn diagram analysis, we found that the FokT group led to the additional downregulation of 2044 genes compared to the shTDP‐43 group (Figure 3G). However, when the differential gene expression profiles of each group were visualized as heatmaps, the overall expression patterns of the FokT and shTDP‐43 groups exhibited high consistency (Figure 3H), and the differences highlighted by the Venn diagram were not significant. Further analysis of the individual heatmap for these 2044 genes revealed that their expression trends were generally similar in the FokT and shTDP‐43 groups, though the changes in the shTDP‐43 group did not reach statistical significance (Figure S1A). Importantly, Gene Ontology (GO) analysis showed that the functional changes caused by the FokT and knockdown groups were nearly identical (Figure S1A–C).

Additionally, we statistically analyzed alternative splicing events in each group, and the results showed that the changes in splicing events between the FokT and shTDP‐43 groups were highly consistent (Figure 3I). To visualize the changes in gene expression, we plotted volcano plots for the NC/FL, NC/FokT, and NC/shTDP‐43 groups. The volcano plots of the NC/FokT and NC/shTDP‐43 groups nearly overlapped (Figure 3K,I), while both differed entirely from the NC/FL group (Figure 3J–L). This further indicates that the effect of FokT is highly similar to the function of TDP‐43 knockdown.

In summary, our results demonstrate that FokT cleaves downstream DNA targets through TDP‐43 dimerization, resulting in a significant reduction in the expression of target genes. This finding highlights the essential role of TDP‐43 dimerization in regulating gene expression and further reveals its physiological functions. In addition, the high degree of similarity in transcriptomic changes between the FokT and shTDP‐43 groups suggests that TDP‐43 is more likely to function as a transcriptional activator in the regulation of these genes. Together, these findings not only uncover the central role of TDP‐43 dimerization in gene regulation but also offer new insights and theoretical support for understanding its pathogenic mechanisms in ALS and other neurodegenerative diseases.

### 2.4 FokT‐seq Technology Precisely Deciphers the DNA Binding Properties and Genomic Distribution of Dimerized TDP‐43

Our study demonstrates that dimerized TDP‐43 can bind DNA, and this binding plays a crucial role in regulating downstream transcription. However, traditional techniques for detecting protein‐DNA interactions, such as ChIP‐seq and Cut&Tag, cannot distinguish between DNA sequences bound by dimerized TDP‐43 and those bound by monomeric TDP‐43. This technical limitation has hindered a deeper understanding of the function of dimerized TDP‐43. Therefore, to specifically detect DNA targets bound exclusively by dimerized TDP‐43, we combined the FokT system with the Guide‐seq technology,^[^ ^25^, ^26^ ^]^ developing a novel method called FokT‐seq.

**Figure 4:**
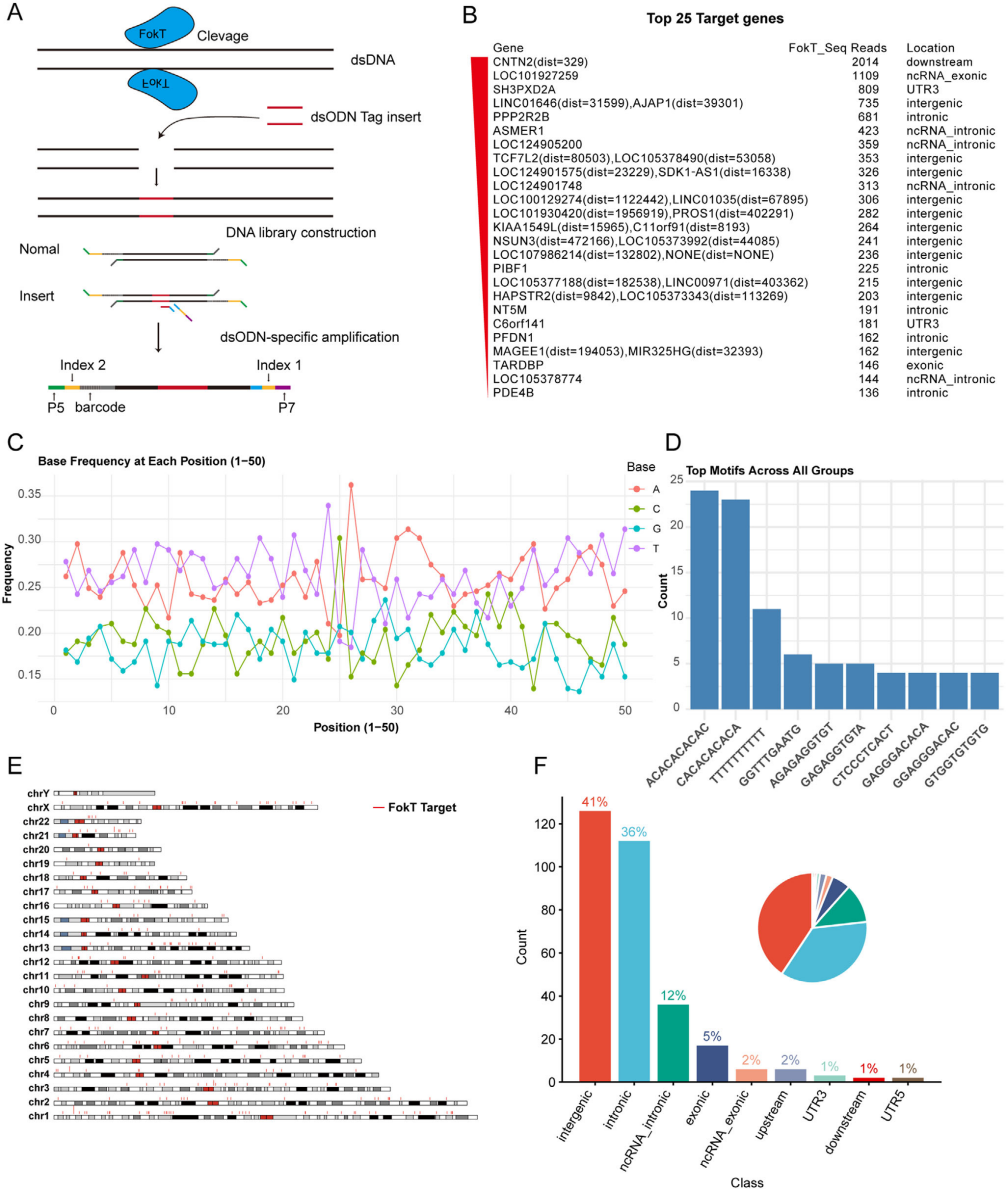
Design and application of the FokT‐seq method. **A**) Schematic overview of the FokT‐seq method. **B**) Top 25 FokT cleavage targets ranked by dsODN read counts. **C**) Line plot showing the nucleotide frequency at each position within the 50 bp sequence region targeted by FokT. **D**) Motif analysis of 311 FokT target sequences, highlighting 10 potential motif sequences. **E**) Ideogram depicting the chromosomal locations of FokT cleavage sites. **F**) Statistical analysis of the genomic locations bound by FokT, presented as a bar graph to indicate the positional preferences of FokT binding.

In the FokT‐seq experiment, we electroporated FokT along with double‐stranded oligodeoxynucleotides (dsODNs) into 293T cells. When dimerized FokT cleaves DNA, the dsODN sequence marks the cleavage sites (Figure **4**A). Subsequently, we performed high‐throughput sequencing of these marked regions and used bioinformatics analysis to precisely identify the locations and frequencies of off‐target sites. Using this approach, we identified 311 DNA sites bound by dimerized TDP‐43 (Table S1, Supporting Information). To visually present these findings, we ranked the top 25 binding sites by dsODN read abundance and listed their genomic locations (Figure 4B). The analysis revealed that dimerized TDP‐43 preferentially binds to intergenic regions.

In the Guide‐seq data analysis, we aligned the reads to the genome to identify regions with continuous read coverage, defining these regions as “windows.” Within each window, the point with the highest read density was considered the potential cleavage site, and a range of ±25 bp around this site was defined as the TDP‐43 binding region. For the 311 identified 50‐bp binding sites, nucleotide composition analysis revealed a significant enrichment of T and A bases (Figure 4C). Motif analysis identified 10 potential DNA sequence motifs that may be bound by dimerized TDP‐43 (Figure 4D).

We further mapped the 311 dimerized TDP‐43 binding sites to the human genome (Figure 4E) and categorized their regional distribution. The results showed that 41% of the binding sites were located in intergenic regions, 36% in intronic sequences, and 12% in the introns of non‐coding RNAs (Figure 4F). These findings provide the first comprehensive analysis of the binding sites of dimerized TDP‐43, revealing its preference for specific genomic regions and offering important biological insights.

More importantly, the FokT‐seq technology not only enables the specific detection of DNA targets bound by dimerized TDP‐43 but also allows for high‐precision genome‐wide localization of these targets. This method provides a powerful tool for investigating the role of dimerized TDP‐43 in gene regulation and offers a new approach for exploring the DNA interactions of other dimerization‐dependent proteins.

### 2.5 FokT‐seq Expands Traditional Databases by Precisely Identifying Novel Targets of Dimerized TDP‐43

**Figure 5:**
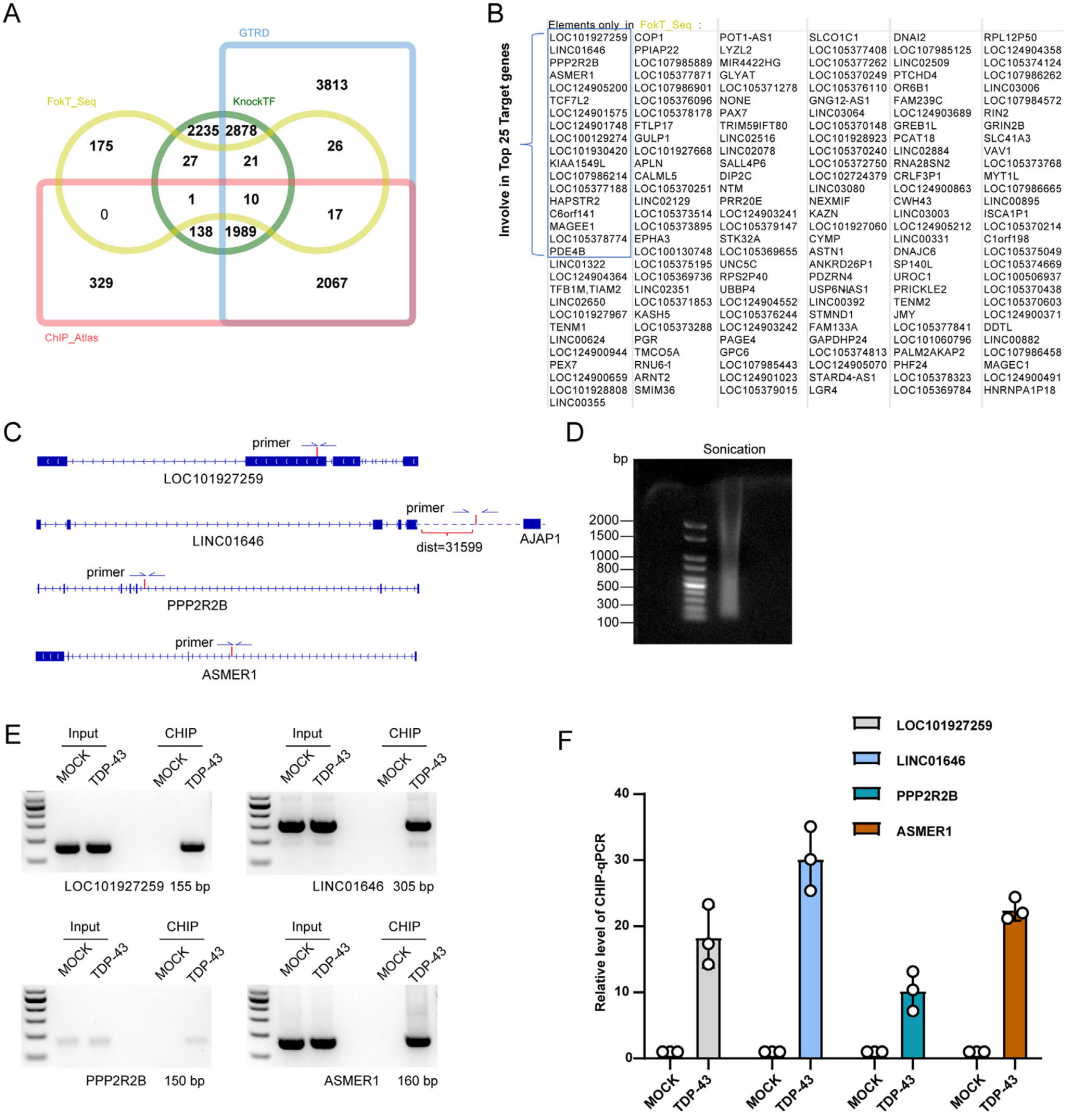
FokT‐seq reveals novel DNA targets of TDP‐43 dimerization. **A**) Venn diagram comparing FokT target sites with existing TDP‐43 target data from KnockTF, CTRD, and ChIP‐Atlas datasets, revealing 175 unique targets not present in known databases. **B**) Display of the 175 unique targets identified in panel a. **C**) Schematic representation showing FokT binding sites on LOC101927259, LINC01646, PPP2R2B, and ASMER1, along with the locations of ChIP‐PCR primers. **D**) Agarose gel electrophoresis results demonstrating that DNA fragments were sheared to sizes between 100–800 bp for ChIP‐PCR quality control. **E**) ChIP‐PCR results confirm that LOC101927259, LINC01646, PPP2R2B, and ASMER1 are bona fide TDP‐43 binding targets. **F**) ChIP‐qPCR quantification demonstrates that TDP‐43 binds to DNA at LOC101927259, LINC01646, PPP2R2B, and ASMER1. The binding locations are consistent with sequencing results. Data are presented as mean ± S.E.M.

After validating the effectiveness of FokT‐seq, we further compared this method directly with three major transcription factor databases to evaluate its unique advantages in identifying binding sites of dimerized TDP‐43. The results showed that among the binding sites detected by FokT‐seq, 102 sites overlapped with targets identified in traditional databases (Figure **5**A). Additionally, we identified 175 unique binding sites that were not detected by conventional methods, and these newly discovered targets were carefully annotated and recorded (Figure 5B).

To further confirm the reliability of these binding sites, we selected four top‐ranked targets—LOC101927259, LINC01646, PPP2R2B, and ASMER1—for validation through ChIP‐PCR experiments (Figure 5C). First, we performed quality checks on the sonicated genomic fragments, which showed that the DNA fragment lengths were primarily distributed between 100–800 bp, meeting the quality standards for ChIP experiments (Figure 5D). Next, the ChIP‐PCR results demonstrated significant binding signals of TDP‐43 at all four targets, further confirming the accuracy and feasibility of the binding sites identified by FokT‐seq (Figure 5E,F).

These findings strongly indicate that FokT‐seq can not only identify known binding sites from traditional databases but also uncover numerous novel targets that are difficult to detect using conventional methods. This technological breakthrough effectively addresses the challenge of distinguishing binding sites of dimerized proteins from those of monomeric proteins, demonstrating significant potential in detecting the DNA‐specific binding of dimerized transcription factors.

More importantly, given the abundance of dimerized proteins in vivo, particularly transcription factors involved in gene expression regulation, the FokT‐seq method provides a powerful new tool for studying the specific interactions between these proteins and DNA. This approach is not only of great significance for research on TDP‐43 but also offers broad application potential for functional analysis and target localization of other dimerized proteins, positioning itself as an essential technique for future studies on transcription factors and their mechanisms of action.

## 3 Discussion

In recent years, accumulating studies have confirmed that TDP‐43 exhibits abnormal aggregation and subcellular localization changes in the neurons of ALS patients.^[^ ^27^, ^28^, ^29^ ^]^ Pathological examinations have shown a significant reduction in nuclear TDP‐43 levels in the motor neurons of ALS patients, accompanied by the presence of misfolded and hyperphosphorylated TDP‐43 aggregates in the cytoplasm, which have been associated with disease pathology.^[^ ^30^ ^]^ Under normal conditions, TDP‐43 predominantly exists as a homodimer; however, in the brains and spinal cords of ALS patients, the level of dimerization is markedly decreased, suggesting that the loss of dimerization may be a critical early event driving TDP‐43 pathology.^[^ ^15^ ^]^ As the stability of TDP‐43 dimerization and its nuclear localization are essential for its functions in RNA splicing,^[^ ^12^ ^]^ transport, and DNA binding regulation, dimerization abnormalities not only impair its roles in post‐transcriptional regulation but may also exacerbate its abnormal cytoplasmic aggregation, ultimately leading to neuronal dysfunction and degeneration.

This study focuses on the role of TDP‐43 dimerization in DNA binding and transcriptional regulation by constructing and validating the FokT system, which combines the FokI catalytic domain with TDP‐43 (Figure 1A). Our experimental results demonstrate that FokT mediates DNA cleavage through TDP‐43 dimerization (Figure 2), with transcriptomic changes induced by FokT showing a 98.4% similarity to those observed upon TDP‐43 knockdown (shTDP‐43) (Figure 3). This highlights the critical role of TDP‐43 dimerization in regulating downstream gene expression and also suggests that TDP‐43 primarily functions as a transcriptional activator. Furthermore, by integrating FokT with Guide‐seq (Figure 4A), we developed the FokT‐seq technology, which enabled the precise identification of DNA binding sites for dimerized TDP‐43. Using FokT‐seq, we discovered numerous novel targets that were undetectable by conventional methods, such as ChIP‐seq and Cut&Tag (Figure 5A). Motif analysis and ChIP‐PCR validation of these newly identified targets further confirmed the feasibility and high efficiency of FokT‐seq in elucidating the binding patterns of dimerized TDP‐43.

Notably, in addition to FokT‐seq, we propose a broader concept and methodological framework called Fok‐seq. Fok‐seq leverages the dimerization‐dependent cleavage property of FokI, enabling its application to other dimerized or oligomerized proteins for the specific detection and high‐throughput localization of DNA binding sites. Given that many transcription factors and regulatory proteins in cells require dimerization (or oligomerization) to bind DNA and exert their functions, Fok‐seq offers a highly feasible and widely applicable approach to study such proteins, overcoming the limitations of existing research tools.

Unlike previous studies that primarily focused on the roles of TDP‐43 in RNA splicing and transport,^[^ ^2^, ^7^, ^31^ ^]^ this study emphasizes its critical function in DNA binding and transcriptional regulation, providing a novel perspective on the disease mechanisms of ALS and other neurodegenerative diseases. Due to the dynamic nature of TDP‐43 dimerization, traditional structural biology methods struggle to capture its stable conformation. By contrast, FokT‐seq and Fok‐seq adopt a functional approach, offering groundbreaking insights into the specific DNA‐binding mechanisms of dimerized proteins. Importantly, FokT‐seq not only aligns with traditional database results but also identifies additional novel binding sites that are challenging to detect using conventional techniques, demonstrating significant advantages in expanding “blind spot” detection.

Future studies could apply FokT‐seq and Fok‐seq to a broader range of cell types, animal models, and clinical samples to investigate the functional differences of dimerized TDP‐43 or other dimerized proteins under various pathological and physiological conditions. Combining these methods with proteomics approaches to study protein‐protein interactions may further elucidate the relationships between dimerized proteins and their cofactors. Additionally, extending Fok‐seq to explore other dimerization‐dependent transcription factors or integrating it with gene‐editing technologies such as CRISPR/Cas holds promise for advancing the mechanistic understanding and therapeutic strategies for neurodegenerative diseases and other related disorders.

In conclusion, this study not only provides new evidence supporting the role of TDP‐43 in ALS pathogenesis but also lays the foundation for a deeper understanding of the central role of dimerized proteins in gene expression regulation.

## 4 Experimental Section

### Plasmid Generation

TDP‐43, FokI tagged with 3×FLAG were generated by amplified the pCI/TDP‐43 and FokI with appropriate primers and cloned into the p3×FLAG‐CMV 7.1. And the FokI‐TDP‐43 was synthesis by inserting TDP‐43 fragment in vector 3×FLAG‐FokI. The FokI‐TDP‐43^N6A^ were generated using the inverse PCR method. shTDP‐43 were constructed by inserting interfering sequences into pLL3.7 (Addgene, Plasmid#11795). Plasmids were constructed using standard cloning procedures and were verified using Sanger sequencing.

### Cell Culture and Transfection

Human embryonic kidney 293T cells (HEK‐293T) were maintained in DMEM (Gibco) supplemented with 15% FBS (Vazyme) at 37 °C in 5% CO_2_. Plasmids were transfected into the cells with Lipofectamine 2000 (Invitrogen) according to the manufacturer's instructions. For shRNA treatments, cells were plated on day 0, transduced with shRNA on day 2 followed by media refresh on day 3, and collected. We performed electroporation using a Neon NxT Electroporation System (Thermo Fisher Scientific) and the Neon Transfection System 10 µl Kit (Thermo Fisher Scientific, cat. no. MPK1025,) at 1500 V, 10 ms and three pulses. A total of 2.5 µg plasmid was used per electroporation, with two electroporations plated per 12‐well plate well. The cells were allowed to incubate in a biosafety hood for 10 min before being transferred to culture plates and returned to a 37 °C incubator.

### Protein‐Protein Cross‐Linking in Cells

Native protein‐protein interactions were stabilized by crosslinking with disuccinimidyl glutarate (DSG; MCE, HY‐114697). In brief, cells grown to 80% confluency in 6 cm plates washed once with phosphate‐buffered saline (PBS), and resuspended in PBS supplemented with EDTA‐free protease inhibitor cocktail (MCE, HY‐K0010), followed by incubation with DSG concentration gradient from 0–400 µM for 30 min at room temperature with gentle rotation. The cross‐linking reaction was quenched by adding 20 mM Tris base (pH7.4) and by incubating for 15 min at room temperature. Cells were collected by centrifugation at 1500 g for 5 min.

### Protein Extraction, Immunoblotting

Cells were lysed with RIPA buffer (Beyotime, P0013B) with EDTA‐free protease inhibitor cocktail (MCE, HY‐K0010) and phosphatase inhibitors (MCE, HY‐K0021). After centrifugation at 12,000 g for 15 min at 4 °C, the supernatant was transferred to new tubes. Nuclear and cytoplasm proteins from 293T cells were prepared using nuclear and cytoplasmic extraction reagents (Biosharp, BL670B). Protein concentrations were determined by Bradford assay (Beyotime, P0011). For reducing SDS‐PAGE, equal amounts of total protein were boiled in SDS sample buffer for 10 min before running in 10% acrylamide gel. Non‐reducing SDS‐PAGE was performed by mixing equal sample with Laemmli sample buffer (BioRad, 161–0737) and subsequent loading on 7.5% acrylamide gel. Proteins were transferred to PVDF (Millipore Sigma, #IPVH00010), and blocked in 5% milk solution in Tris‐buffered saline (TBS) for 1 h. Membranes were incubated with primary antibodies diluted in Blocking Buffer overnight at 4 °C. Membranes were washed in TBST (0.1% Tween in TBS) three times, 5 min each, incubated with secondary antibodies conjugated to HRP diluted in Blocking Buffer for 1 h (1:5000, Abclonal), washed with TBST, visualized by the ECL western blotting substrate, and detected by ChemiScope 6100 (Clinx). Specific immunosignals were quantified by using the Fuji imageJ Wiki software.

### Immunofluorescence Staining

HEK‐293T cells grown on coverslips in a 24‐well plate were transfected and treated as described above. The cells were fixed in 4% paraformaldehyde in PBS for 15 min at room temperature, and the cells were permeabilized in 0.5% Triton X‐100 (Biosharp, BS084) in PBS for 15∼30 min and blocked with 3% goat serum in PBST (0.1% Triton X‐100 in PBS) for 1 h at RT. The above primary and secondary antibodies were then incubated in the blocking buffer at 4 °C overnight or for 48 h, or at RT for 1–2 h. After 3 washes with PBST, cells were mounted on glass slides using Mounting Medium with DAPI sealer (ZSGB‐Bio, ZLI9557). The immunofluorescence was revealed with a Zeiss Laser Confocal Microscope LSM800.

### Comet Assay

The comet assay was performed according to the Comet Assay Kit (Beyotime, C2041S). Briefly, HEK‐293T cells were cultured in a 6‐well plate. Cells were then collected, resuspended at single‐cell status by 1× PBS with an additional 1:10 ratio of 1% low‐melting‐point agarose and applied to a 1% agarose precoated slide for electrophoresis using the alkaline electrophoresis solution. Finally, fluorescent nucleic acid staining solution was dropped onto a slide for visualization of the DNA breaks under fluorescence microscopy and the quantifications were processed by the software Opencomet.

### RNA Sequencing

HEK293T cells were transfected with plasmids expressing 3×FLAG‐TDP‐43, 3×FLAG‐NLS‐FokT, or shTDP‐43, respectively, using [insert transfection reagent, e.g., Lipofectamine 3000] according to the manufacturer's protocol. Cells were incubated for 72 h post‐transfection under standard culture conditions (37 °C, 5% CO_2_, in DMEM supplemented with 10% FBS). Total RNA was then extracted using [insert RNA extraction kit, e.g., TRIzol reagent] following the manufacturer's instructions. RNA integrity and quality were assessed using an Agilent Bioanalyzer 2100 to ensure RNA integrity numbers (RIN) > 7.

For transcriptome profiling, RNA sequencing (RNA‐Seq) was performed by BGI Genomics. Libraries were prepared using [insert library preparation kit, e.g., NEBNext Ultra II RNA Library Prep Kit] and sequenced on an [insert sequencing platform, e.g., MGISEQ‐2000] to generate paired‐end reads with a sequencing depth of 10 gigabases (Gb) per sample. The resulting raw sequencing data underwent quality control using [insert software, e.g., FastQC], and high‐quality reads were aligned to the reference genome [insert genome version, e.g., GRCh38] using [insert alignment tool, e.g., STAR].

### Guide‐seq

HEK‐293T cells were electroporated with a combination of double‐stranded oligodeoxynucleotides (dsODNs) and either the CMV7.1 plasmid or the 3×FLAG‐NLS‐FokT plasmid using [insert electroporation system, e.g., Lonza Nucleofector II] according to the manufacturer's instructions. Cells were incubated for 72 h post‐electroporation under standard culture conditions (37 °C, 5% CO_2_, in DMEM supplemented with 10% FBS).

Genomic DNA was extracted using [insert DNA extraction kit, e.g., Qiagen DNeasy Blood & Tissue Kit] following the manufacturer's protocol. The extracted DNA was then sent to Generulor BioTechnology Co., Ltd. (Zhuhai, China) for GUIDE‐Seq library preparation and sequencing analysis. GUIDE‐Seq libraries were constructed using the [insert library preparation protocol, if available] and sequenced on an [insert sequencing platform, e.g., Illumina NovaSeq 6000] to obtain high‐quality paired‐end reads.

Sequencing data were analyzed to identify off‐target cleavage events. Raw reads were processed using [insert software/tool, e.g., GUIDE‐seq pipeline], and aligned to the human reference genome [insert genome version, e.g., GRCh38] to map cleavage sites. Subsequent analyses focused on the identification and quantification of specific on‐target sites.

### Chromatin Immunoprecipitation (ChIP) Assay

Chromatin immunoprecipitation (ChIP) assay was performed according to the ChIP Chromatin Immunoprecipitation Kit (GENE CREATE, JKR23002A). Briefly, crosslinked chromatin was sonicated into 200‐ to 1000‐bp fragments. Anti‐TDP‐43 antibody was used to precipitate DNA‐protein complexes. Normal rabbit immunoglobulin G (IgG) was used as a negative control. ChIP‐derived DNA was quantified using PCR. The primers are listed in Table S2 (Supporting Information).

### Statistical Analysis

Statistical analysis was performed using GraphPad Prism 8.0 software (GraphPad Software Inc., San Diego, CA, USA). Data are presented as mean ± S.E.M., and were statistical analyzed by two‐tailed *t*‐test (for comparison of two groups) or one‐way ANOVA. followed by Holm‐Šídák's Multiple comparisons post‐hoc test (for comparison of more than two groups). When at least one group was not normally distributed, differences between groups were tested using Mann–Whitney test (for comparison of two groups) or Kruskal–Wallis, followed by a Dunn's Multiple comparisons post‐hoc test (for comparison of more than two groups). A value of *p* ≤ 0.05 was considered statistically significant. The following code was used to indicate the level of significance: *p≤ 0.05; **p≤ 0.01; ***p≤ 0.001; ****p≤ 0.0001.

## Conflict of Interest

The authors declare no conflict of interest.

## Author Contributions

M.Y., Q.W. and R.Y. contributed equally to this work. M.Y., Q.W., and R.Y. performed the experiments. M.Y. designed and supervised the experiments, analyzed and interpreted the results, and wrote the original manuscript. X.W. and J.G. supervised the experiment, analyzed and interpreted the results, edited and finalized the manuscript . All authors reviewed and approved the final version of the manuscript.

## Supporting information



Supplemental Table S1

Supplemental Table S2

Supplemental Table S3

Supplemental Material

## Data Availability

The data that support the findings of this study are available from the corresponding author upon reasonable request.
