# Supplementary material for: Dimer‐Specific FokT‐seq Reveals DNA‐Binding Dimerization and Novel Genomic Targets of TDP‐43
Source: Adv Sci (Weinh). 2025 Aug 23;12(42):e08902. doi: 10.1002/advs.202508902 (PMC12622432; doi:10.1002/advs.202508902)
Supplement: Supplementary file 1 — Supplemental Table S1 [file ADVS-12-e08902-s001.docx]

**Supplementary Material**

**Dimer-specific FokT-seq Reveals DNA-binding Dimerization and Novel Genomic Targets of TDP-43**

Mingming Yang^1,2,3^†^·^, Qi Wang^1^†, Ruolan Yan^2^†, Xiaochuan Wang^1,3*^, Jianlan Gu^2*^

^1^Department of Pathophysiology, School of Basic Medicine, Key Laboratory of Education Ministry/Hubei Province of China for Neurological Disorders, Tongji Medical College, Huazhong University of Science and Technology, Wuhan, 430030, China

^2^Department of Biochemistry and Molecular Biology, School of Medicine, Key Laboratory of Neuroregeneration and Ministry of Education of Jiangsu, Co-innovation Center of Neuroregeneration, Nantong University, Nantong, 226001, China

^3^Hubei Key Laboratory of Cognitive and Affective Disorders, Institute of Biomedical Sciences, School of Medicine, Jianghan University, Wuhan, 430056, China

†The authors contributed equally to this work

*Corresponding authors: Jianlan Gu (lead contact), E-mail: ntgjlan@ntu.edu.cn; Xiaochuan Wang, [wangxiaochuan@hust.edu.cn](mailto:wangxiaochuan@hust.edu.cn);

**
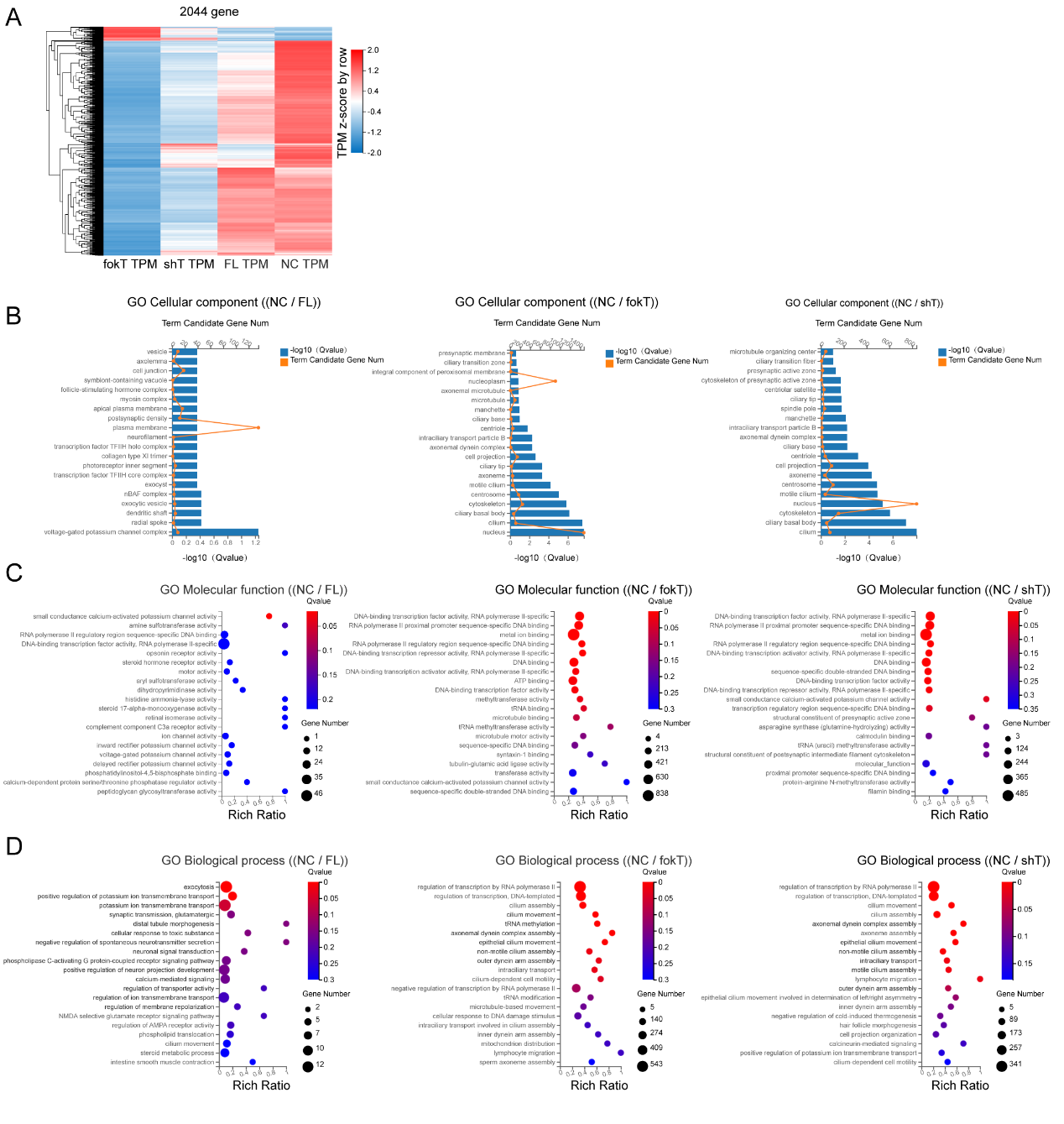
**

**Supplementary Fig.1 FokT and TDP-43 Knockdown Induce Similar Biological Functional Changes. A**) Heatmap displaying the 2044 FokT-specific genes. Normalization was performed using the z-score method (row direction). The X-axis represents the log2-transformed expression values (log2[TPM+1]) for samples, and the Y-axis represents genes. In the default color scheme, red indicates higher expression levels, while blue indicates lower expression levels. **B**) Bar graph illustrating GO enrichment analysis. The lower X-axis represents the enrichment ratio (calculated as: Rich Ratio = Term Candidate Gene Num / Term Gene Num), and the upper X-axis represents the number of differentially expressed genes (DEGs) annotated to each GO term. The Y-axis represents GO terms, and the blue bars indicate enrichment significance (Q-value). **C-D**) Bubble plots for GO enrichment analysis. The X-axis represents the enrichment ratio (Rich Ratio = Term Candidate Gene Num / Term Gene Num), and the Y-axis represents GO terms. The size of the bubbles indicates the number of DEGs annotated to each GO term, while the color represents enrichment significance (Q-value). A more red color indicates a smaller Q-value, reflecting higher enrichment significance.
