# Supplementary material for: Dimer‐Specific FokT‐seq Reveals DNA‐Binding Dimerization and Novel Genomic Targets of TDP‐43
Source: Adv Sci (Weinh). 2025 Aug 23;12(42):e08902. doi: 10.1002/advs.202508902 (PMC12622432; doi:10.1002/advs.202508902)
Supplement: Supplementary file 3 — Supplemental Table S3 [file ADVS-12-e08902-s004.docx]

**Table S2 Antibody used for WB/IF in this study**

| Antibody | Type | Species | Specificity | Source (Catalog No.) | Usage | Dilution |
| --- | --- | --- | --- | --- | --- | --- |
| Anti-TDP-43 | Poly | R | TDP-43 | ProteinTech (10782–2-AP) | WB | 1:10,000 |
| Anti-β-ACTIN | Mono | R | β-ACTIN | ABclonal (AC038) | WB | 1:10,000 |
| Anti-FLAG | Poly | R | FLAG | ProteinTech (20543-1-AP) | WB | 1:5000 |
| 53BP1 | Mono | R | 53BP1 | CST (88439) | IF | 1:500 |
| Lamin B1 | Poly | R | Lamin B1 | ABclonal (A1910) | WB | 1:1000 |
| p-Histone H2AX-S139 | Mono | R | YH2A.X (S139) | ABclonal (AP0687) | WB/IF | 1:1000/1:200 |
| GAPDH | Mono | M | GAPDH | ABclonal (AC033) | WB | 1:8000 |
