# Supplementary material for: Dimer‐Specific FokT‐seq Reveals DNA‐Binding Dimerization and Novel Genomic Targets of TDP‐43
Source: Adv Sci (Weinh). 2025 Aug 23;12(42):e08902. doi: 10.1002/advs.202508902 (PMC12622432; doi:10.1002/advs.202508902)
Supplement: Supplementary file 4 — Supplemental Material [file ADVS-12-e08902-s002.docx]

**Table S3 Primers used for qPCR/RT-PCR in this study**

| ***CHIP-PCR***  ***GENE*** | **Primers (5’→3’)** | |
| --- | --- | --- |
|  | **Forward** | **Reverse** |
| *LOC101927259* | AAAAAAGCATCCAGATTTAGAACTTGACAGG | AATTGCTTAACGCCCAGGTTTTTTATCCC |
| *LINC01646* | CAAACTGCTGGCATCGTCCTC | CGCCCTGAGAGACAAGGAAAATGG |
| *PPP2R2B* | TCAATGAAGCTTAGAAAAGCTTCATGGAA | TTATAATCTGAGGACCTAGCATAGGGACTG |
| *ASMER1* | GTAACTATCAGTTTCCTAGGGCTGCCA | GGGGCAAGGAAGAATCTCTCAGAAACGTAA |
